# Assessing the Presence of Post-Traumatic Stress and Turnover Intention Among Nurses Post–Middle East Respiratory Syndrome Outbreak: The Importance of Supervisor Support

**DOI:** 10.1177/2165079919897693

**Published:** 2020-03-09

**Authors:** Heeja Jung, Sun Young Jung, Mi Hyang Lee, Mi Sun Kim

**Affiliations:** 1 Konyang University; 2 Seoul Health Foundation

**Keywords:** post-traumatic stress disorder, turnover intention, supervisor support, nurses

## Abstract

*Background:* South Korea faced the Middle East Respiratory Syndrome (MERS) outbreak for the first time in 2015, which resulted in 186 infected patients and 39 deaths. This study investigated the level of post-traumatic stress disorder (PTSD) and turnover intention, the relationship between PTSD and turnover intention, and the buffering effect of supervisor support among nurses post-MERS outbreak. *Methods*: In total, 300 nurses from three of 15 isolation hospitals in South Korea were invited to participate. We collected data pertaining to PTSD, turnover intention, supervisor support, work-related factors, and socio-demographic factors through a structured survey distributed to the nurses at the hospitals after the outbreak. For the statistical analyses, descriptive statistics and multiple regression were employed. *Findings*: Of the 147 participants, 33.3% were involved in the direct care of the infected patients, whereas 66.7% were involved in the direct care of the suspected patients. More than half (57.1%) of the nurses experienced PTSD, with 25.1% experienced full PTSD and 32.0% with moderate or some level of PTSD. The mean score of turnover intention was 16.3, with the score range of 4 to 20. The multiple regression analysis revealed that PTSD was positively associated with turnover intention, and supervisor support had a strong buffering effect. *Conclusion/Application to Practice*: These findings confirmed that after a fatal infectious disease outbreak like MERS, nurses experience high level of PTSD and show high intention to leave. Organizational strategies to help nurses to cope with stress and to prevent turnover intention, especially using supervisor support, would be beneficial.

## Introduction

Middle East Respiratory Syndrome (MERS) is a viral respiratory disease caused by novel coronavirus, which was first occurred in Saudi Arabia in 2012 (World Health Organization [WHO], 2018). As of September 2019, there were 2,468 confirmed patients globally and 851 deaths (WHO, 2019). South Korea faced the MERS outbreak for the first time in May 2015, and the end of the outbreak was declared on July 7, 2015 (Korea Centers for Disease Control & Prevention [KCDC], 2018). Overall, there were 186 infected patients and 39 deaths in 15 cohort isolation hospitals.

Among the 186 total infected patients, 44.1% (*n* = 82), 34.9% (*n* = 65), and 21.0% (*n* = 39) were pre-admitted patients, family members or visitors, and health care professionals, respectively. Out of the 39 infected health care professionals, 15 were nurses. Due to the lack of vaccine or special treatment along with a high mortality rate (36%), nurses directly involved in the care of MERS patients showed high levels of stress and fear (Khalid et al., 2016; WHO, 2018). Similarly, Taiwanese nurses who were directly involved with the care of patients of Severe Acute Respiratory Syndrome (SARS) in 2003 reported more severe post-traumatic stress compared with the nurses who were not involved in the direct care (Chen et al., 2005).

Post-traumatic stress disorder (PTSD) is associated with exposure to traumatic events resulting in a state of psychological unbalance (Blake et al., 1995). Medical staff, including nurses, are more likely to be in environments that increase their sensitivity to PTSD (Tang et al., 2017; Wu et al., 2009). Another consistent issue of the nursing industry is the workforce shortage and high turnover rates. Particularly in Korea, less than 50% of registered nurses are actively working, and the average turnover rate is 12.4%, with a rate of 34% for newly graduated nurses (Ministry of Health and Welfare, 2018). Prior studies on the relationship between post-traumatic stress and turnover intention reported strong associations among nurses working in emergency rooms (Han & Lee, 2013; Maeng & Sung, 2015). Many other factors, including socio-demographic factors, organizational commitment, perceived advancement opportunities, job and career satisfaction, salary level, and workplace relationships and support, have also been found to influence turnover intention (Ayalew et al., 2015; Brunetto et al., 2013; Y. Kim & Kang, 2015; Laschinger, 2012; Oyeleye et al., 2013; Takase et al., 2016). However, workplace relationships and support, especially supervisor support, have been proven to be strong buffers on work-related traumatic stress, work-related stress, and turnover (LaRocco et al., 1980; Stephens & Long, 2000); supervisor support has shown to be positively associated with job and career satisfaction (Nissly et al., 2005).

To date, there is a lack of studies investigating the relationship between post-traumatic stress and turnover intention among nurses in the event of outbreak epidemics, such as MERS. The purpose of this study was to examine the levels of PTSD and intention to leave among a sample of Korean nurses who were directly involved in the care during the MERS outbreak, as well as the buffering effects of supervisor support on this relationship.

## Methods

We conducted a cross-sectional study, in which quantitative data were collected through a survey from October 1 through November 30, 2015, shortly after the MERS epidemic ended. During the 2015 MERS outbreak, 15 hospitals were designated as cohort isolation facilities. We contacted all hospitals and three agreed to participate in the study. These included two hospitals in Daejeon city that were isolated for infection control from June 9 through June 23, and the third hospital in Gyeonggi province which was isolated from May 31 through June 15 (KCDC, 2018). The KCDC (2018) announced the end of the cohort isolations on July 26. We conducted the surveys approximately 2 months after the announcement; we considered that the nurses have recovered to normal working conditions by this time. The nurses were asked to recall during the period of the MERS outbreak and answer the survey questionnaires based on that prior time period.

A total of 300 nurses from three general hospitals, who provided either direct or indirect care for MERS, were invited to participate in the study. The participants included in the final analysis were those who provided direct care during the MERS outbreak. This study was conducted after receiving the Institutional Review Board approval from the Konyang University Hospital (IRB KYUH 2015-09-009-001) as well as informed consent from the participants.

### Data Collection

Post-traumatic stress disorder was assessed by using the Impact of Event Scale–Revised Korean version (IES-R-K; Eun et al., 2005). The original Impact of Event Scale–Revised (IES-R; Weiss & Marmar, 1997) was composed of three sub-dimensions (avoidance, intrusion, and hyperarousal) consisting of 22 questions, and the IES-R-K is composed of five sub-dimensions (avoidance, intrusion, hyperarousal, and sleep problems and numbness). The IES-R-K consists of 22 questions including six items on hyperarousal (score range of 0–24), six items on avoidance (score range of 0–24), five items on intrusion (score range of 0–20), and five items on sleep problems and numbness (score range of 0–20). The 5-point Likert-type scale (0–4 points) consists of the total sum of scores for the four sub-dimensions ranging from 0 to 88, with higher scores indicating higher level of post-traumatic stress. Eun et al. (2005) presented the total PTSD score of 25 as the cutoff point for experiencing full PTSD, and the score of 18 to 24 indicating moderate or some level of PTSD. The Cronbach’s alpha of the original IES-R was .79, and that of the IES-R-K was .93 (Eun et al., 2005; Weiss & Marmar, 1997). The Cronbach’s alpha of the IES-R-K in this study was .95. The nurses were asked to recall their feelings and emotions associated with the MERS outbreak time period.

Supervisor support was measured using the Korean version of the Job Content Questionnaire (K-JCQ; Eum et al., 2007). The K-JCQ consists of three sub-domains (autonomy of work, job demands, and social support of the workplace), with a total of 45 items. The social support domain of the K-JCQ includes supervisor support, co-worker support, and job instability. In this study, we used four questions regarding the supervisor support, including questions 34 (“My boss is interested in the welfare of his or her subordinates”), 35 (“My boss is interested in my opinion and listens carefully.”), 37 (“My boss helps me to accomplish my tasks.”), and 38 (“My boss is good at making people work together”). A 4-point Likert-type scale (1 = *strongly disagree*, 2 = *disagree*, 3 = *agree*, 4 = *strongly agree*) was used. The total possible score ranges between 4 and 19, with higher scores indicating higher supervisor support. Cronbach’s alpha was .84 at the time of development of the measurement (Karasek et al., 1998) and that of the K-JCQ (Eum et al., 2007) was .71; the Cronbach’s alpha in this study was .93.

Turnover intention was estimated by the turnover intention measurement (H. S. Park, 2002), which is the Korean version of the scale originally developed by Lawler (1983). The measurement consists of four items, each of which is a 5-point Likert-type scale (1 = *very unlikely*, 2 = *unlikely*, 3 = *neutral*, 4 = *likely*, 5 = *very likely*). The possible range of scores is from 4 to 20, with higher scores indicating higher intention to leave. The Cronbach’s alpha for the original measurement was .88 (H. S. Park, 2002) and that of the current study was .84.

The General Health Questionnaire (GHQ) was used to measure current self-reported mental health. We used the GHQ-12, a reliable and sensitive short form which is ideal for research studies (Liang et al., 2016). The scale consists of 12 items and is a 4-point Likert-type scale (0 = *much less than usual*, 1 = *same as usual*, 2 = *more than usual*, 3 = *much more than usual*), with a total score ranging from 1 to 36. The cutoff point in the scores of GHQ-12 is 24, indicating the presence of mental health problems (Makowska et al., 2002). The GHQ was included as one of the confounding variables.

To measure the difference of stress levels between the period of the MERS outbreak and after the outbreak, nurses were asked to recall their stress levels during the MERS outbreak and compare it with their current day-to-day stress levels after the outbreak. A question asking the stress level during the outbreak in a 5-point Likert-type scale and another question asking the stress level after the outbreak were included in the survey. The authors, then, calculated the difference between the two levels of the stress.

### Data Analysis

Data were analyzed using the Statistical Package for Social Sciences, Version 20.0 (SPSS, Inc., 2011). The descriptive statistics were calculated using frequency and percentage, as well as means and standard deviation. Cross-tabulation, including *t* test and *F* test, was conducted to examine the characteristics of participants according to PTSD, supervisor support, and turnover intention measures. The relationship between PTSD (independent variable) and turnover intention (dependent variable), as well as the buffering effect of supervisor support as an effect modifier, was examined by multiple regression analysis. Work experience, work position, shift work, department, marital status, level of education, annual income, mental health status, difference in stress levels, and level of involvement in the care of MERS patients were considered as covariates (Jeong et al., 2008; Mosadeghrad, 2013; Yang & Kim, 2016).

## Results

Table 1 describes the general characteristics of the study participants. Of the 300 invited participants, 152 responded with 147 providing usable data (response rate: 49%).

**Table 1.** Demographic Characteristics of Nurses Who Worked During an MERS Outbreak (*N* = 147)

Variables	*N* or *M*	% or *SD*
Work experience		
<1 year	27	18.4
1–4 years	77	52.4
5–9 years	28	19.0
>10 years	15	10.2
Work position		
Staff nurse	134	91.2
Charge nurse and head nurse	13	8.8
Shift work		
No	3	2.0
Yes	144	98.0
Department		
Intensive care	27	18.4
Emergency services	27	18.4
General	92	62.6
Others	1	0.7
Marital status		
Single	126	85.7
Married	21	14.3
Level of education		
3-year college	51	34.7
4-year college or higher	96	65.3
Annual income (US$)		
<30,000	92	62.6
≥30,000	55	37.4
Mental health: GHQ^a^	24.24	7.29
Stress (difference between the period of outbreak vs. every day)	1.34	1.88
Level of involvement during the MERS outbreak		
Directly involved with the treatment of infected patient	49	33.3
Directly involved with the treatment of suspected patient	98	66.7
PTSD		
Full PTSD (scores of 25 and higher)	37	25.1
Moderate/some PTSD (scores 18–24)	47	32.0
No PTSD (scores 0–17)	63	42.9
Supervisor support^a^	10.82	2.16
Turnover intention^a^	16.29	3.33

*Note.* MERS = Middle East Respiratory Syndrome; GHQ = General Health Questionnaire; PTSD = post-traumatic stress disorder.

a Means and standard deviations are present.

All of the participants were female, of which 52.4% had work experience of 1 to 4 years, 18.4% with less than 1 year, 19.0% with 5 to 9 years, and 10.2% with over 10 years of work experience. The majority were staff nurses (91.2%) who worked in rotating shifts (98%). Approximately 37.0% of the nurses worked in either intensive care or in emergency services, whereas 62.6% of them worked in general medicine departments. The majority of the nurses were single (85.7%) and completed 4 years of college or higher (65.3%), and earned an annual salary of more than US$30,000 (62.6%).

Of the 147 nurses, 33.3% of them were directly involved with the treatment of confirmed infected patients, whereas 66.7% of them were directly involved with the treatment of suspected patients. The mental health of the nurses, measured by GHQ, showed the mean score of 28.2 (of 36), indicating problematic self-rated mental health. The difference of stress levels between every day and the days during the outbreak was 1.34, which showed that the stress level during the outbreak was higher.

A total of 57.1% (*n* = 84) of the nurses experienced PTSD. Considering the score of 25 and above as experiencing full PTSD, 25.1% (*n* = 37) experienced full PTSD and 32.0% (*n* = 47) experienced some level of PTSD, with scores of 18 to 24. The mean score of supervisor support was 10.8 with the range of 4 to 19, which indicated moderate supervisor support. The turnover intention score ranged from 4 to 20, and the mean turnover intention in this study was 16.3, which was high.

Table 2 shows the characteristics of participants according to the PTSD, supervisor support, and turnover intention measures. Department (*p* < .05), mental health (*p* < .01), and the level of involvement during the MERS outbreak (*p* < .05) were associated with post-traumatic stress. Mental health was negatively associated with supervisor support (*p* < .01). Years of work experience (*p* < .01), shift work (*p* < .05), marital status (*p* < .05), annual income (*p* < .01), and self-reported mental health (negative association, *p* < .01) were associated with turnover intention.

**Table 2.** Measures of PTSD, Supervisor Support, and Turnover Intention Among Nursing Staff (*N* = 147)

Confounders	Main variables								
	PTSD			Supervisor support			Turnover intention		
	*M* ± *SD*	*t* or *F*	*p* value	*M* ± *SD*	*t* or *F*	*p* value	*M* ± *SD*	*t* or *F*	*p* value
Work experience		0.088	.088		1.282	.283		4.207	.007
<1 year	29.19 ± 9.31			11.37 ± 1.94			14.70 ± 4.36		
1–4 years	29.58 ± 9.97			10.74 ± 2.04			17.10 ± 2.41		
5–9 years	30.25 ± 9.93			10.32 ± 2.42			16.04 ± 3.60		
>10 years	28.87 ± 6.88			11.20 ± 2.54			15.47 ± 3.76		
Work position		1.331	.251		1.251	.265		1.458	.229
Staff nurse	29.28 ± 9.35			10.76 ± 2.13			16.40 ± 3.24		
Charge nurse and head nurse	32.46 ± 10.87			11.46 ± 2.44			15.23 ± 4.15		
Shift work		0.011	.918		1.506	.222		4.444	.037
No	29.00 ± 7.55			12.33 ± 1.53			12.33 ± 6.66		
Yes	29.58 ± 9.55			10.79 ± 2.16			16.38 ± 3.21		
Department		3.178	.026		1.023	.384		0.696	.556
Intensive care	34.44 ± 12.66			10.30 ± 2.57			16.19 ± 3.66		
Emergency services	28.15 ± 8.82			11.30 ± 2.55			15.63 ± 2.91		
General	28.62 ± 8.22			10.85 ± 1.89			16.54 ± 3.35		
Others	23 ± 8.10			10 ± 1.98			14 ± 2.35		
Marital status		0.388	.534		2.265	.135		5.345	.022
Single	29.37 ± 9.61			10.71 ± 2.16			16.55 ± 3.19		
Married	30.76 ± 8.88			11.48 ± 2.04			14.76 ± 3.78		
Level of education		3.235	.074		1.655	.2		0.463	.497
3-year college	27.65 ± 7.62			10.51 ± 2.33			16.55 ± 2.93		
4-year college or higher	30.58 ± 10.25			10.99 ± 2.05			16.16 ± 3.52		
Annual income (US$)		0.216	.643		0.244	.622		7.443	.007
<30,000	29.28 ± 9.30			10.89 ± 1.82			16.86 ± 2.74		
≥30,000	30.04 ± 9.88			10.71 ± 2.64			15.35 ± 3.97		
Level of involvement during MERS outbreak		6.756	.010		0.455	.501		3.723	.056
Directly involved with the treatment of infected patient	32.39 ± 11.20			10.65 ± 2.50			15.55 ± 4.01		
Directly involved with the treatment of suspected patient	28.15 ± 8.22			10.91 ± 1.97			16.66 ± 2.88		
Mental health: GHQ	Coefficient of correlation: .389**			Coefficient of correlation: –.176**			Coefficient of correlation: –.420**		
Stress (difference between outbreak vs. every day)	Coefficient of correlation: –.024			Coefficient of correlation: –.043			Coefficient of correlation: –.062		

*Note.* PTSD = post-traumatic stress disorder; MERS = Middle East Respiratory Syndrome; GHQ = General Health Questionnaire.

** *p* < .01.

Table 3 shows the results from the multiple regression analysis in which we observed that nurses with work experience of between 1 and 4 years (β = .313), direct involvement with the treatment of a suspected patient (β = .224), and high score of PTSD (β = .188) were positively associated with higher intention to leave. Higher supervisor support (β = −.395) was, on the contrary, associated with lower turnover intention. The interaction between PTSD and supervisor support was significant (β = .177) with intention to leave, illustrating the buffering effect of supervisor support.

**Table 3.** Multiple Regression Analysis Predicting Intent to Leave Among Nursing Staff Employed During MERS Outbreak

Predictors	Model 1		Model 2		Model 3	
	β	*p*	β	*p*	β	*p*
Work experience (1–4 years)	.379	<.000	.327	.001	.313	.002
Work experience (5–9 years)	.167	.097	.101	.284	.073	.435
Work experience (>10 years)	.082	.371	.073	.394	.065	.441
Directly involved with the treatment of a suspected patient	.220	.006	.221	.003	.224	.003
PTSD	.206	.010	.132	.081	.188	.017
Supervisor support			−.362	<.000	−.395	<.000
PTSD × Supervisor Support					.177	.024
Changes in *F*	5.085***		23.538***		5.243*	
Adjusted *R*^2^	.123		.244		.266	

*Note.* MERS = Middle East Respiratory Syndrome; PTSD = post-traumatic stress disorder.

* *p* < .05. ***p < .001.

## Discussion

The purpose of this study was to examine the levels of PTSD and turnover intention of nurses after the MERS outbreak in Korea. The result showed that 57.1% of the nurses who were involved with the direct care of either infected or suspected MERS patients experienced PTSD. In particular, 25.1% of the nurses experienced full level of PTSD, which is higher than 20.4% of nurses experiencing full level of PTSD using the same IES-R-K from a study on nurses working at emergency departments (Han & Lee, 2013). Another study on Japanese firefighters using the original IES-R measure showed that only 9.7% of the participants experienced full level of PTSD (Saijo et al., 2012). A study of medical staff involved with patient care during the SARS outbreak found that 10% of the respondents had experienced high levels of PTSD (Wu et al., 2009). Another study found that around 20% of participating doctors and nurses showed PTSD symptoms that were involved in the care of Avian influenza A (H7N9) patients during the H7N9 influenza epidemic in 2015 to 2016 (Tang et al., 2017). The average turnover intention of nurses in this study was 16.29 when the total score range is from 4 to 20, showing high turnover intention. A previous study that used the same measurement revealed average score of 15.41 among nurses working at emergency department (Lee & Ahn, 2015). Other studies also showed lower scores compared with that of our research, which were 13.75 among nurses working at general hospitals (C.-H. Kim et al., 2009) and 9.72 among nurse managers (K. O. Park et al., 2012).

Additional aim of this study was to investigate the relationship between PTSD and turnover intention. The result of this study validated significant and positive relationship between PTSD and turnover intention among nurses who were involved with the care during the MERS outbreak. Prior studies on nurses have presented the same results. A study on Korean nurses working at emergency departments revealed significant and positive association between PTSD and turnover intention (Han & Lee, 2013). Also, after a traumatic incident, more than 20% of nurses working at emergency departments displayed high turnover intention.

The final investigation was to see whether supervisor support has buffering effect between PTSD and turnover intention, which was proven to be legitimate. Social support of workplace, including supervisor support, has been known to act as a moderator against the impact of high stress on well-being and related health outcomes on the buffering hypothesis of Cohen and Wills (1985). Other study has investigated the buffering effect of social support and higher level of organizational stress as well as turnover intention (Nissly et al., 2005). However, majority of studies on buffering effect of social support studies were done on the relationship between general or job stress and turnover intention, so future studies should be specified on the relationship between PTSD and turnover intention during fetal epidemic events as well as the buffering effect of social support, such as supervisor support.

### Strengths and Limitations

Despite the fact that this is one of the few studies on nurses who were involved with the direct patient care during the MERS outbreak in Korea, it has some limitations. Fifteen hospitals were cohort isolated during the MERS breakouts; however, only three hospitals agreed to participated in the study. The small sample size limits our ability to generalize the findings beyond the study hospitals. In addition, the three hospitals that agreed to participate are those that were praised by the media for their successful response to the pandemic. Therefore, the levels of PTSD and the buffering effect of supervisor support might have different results if all of 12 hospitals participated in the research. In addition, data were collected after the outbreak due to facility isolation during the outbreak. Post-traumatic stress disorder, turnover intention, and supervisor supports were all asked as recall questions 2 months after the outbreak, introducing possible recall bias.

### Implication for Occupational Health Nursing

The results of this study are significant and relevant because the participants were nurses who were involved with direct patient care during the MERS outbreak. More than half (57.1%) of the nurses experienced PTSD—25.1% with full level and 32.0% with some level of PTSD. Also, PTSD and turnover intention were significantly and positively related. In addition, the results of this study suggest that social support, particularly supervisor support, should be provided to reduce the impact of PTSD on nurses’ turnover intentions in the case of serious infectious diseases. Support system should be discussed at departmental, organizational, and national levels. Infectious disease and occupational health professionals should consider developing and implementing coping management strategies to reduce PTSD and turnover intention related to epidemic outbreaks as well as providing educational programs to supervisors to provide adequate support to nurses.

**Applying Research to Practice**

Among 147 nurses who were involved with the direct care of either infected or suspected Middle East Respiratory Syndrome (MERS) patients, 25.1% experienced full post-traumatic stress disorder (PTSD) and 32.0% experienced moderate or some level of PTSD. Turnover intention was also high among this cohort. High PTSD scores were positively associated with high turnover intention, and supervisor support was proven to have a strong buffering effect. These findings suggest that supervisor support is a successful management strategy to lower turnover intention post epidemic outbreak. Organizational strategies to help nurses to cope with stress and to prevent turnover intention, especially using supervisor support, would be beneficial.
